# Supplementary material for: Shifts in methanogenic archaea communities and methane dynamics along a subtropical estuarine land use gradient
Source: PLoS One. 2020 Nov 24;15(11):e0242339. doi: 10.1371/journal.pone.0242339 (PMC7685437; doi:10.1371/journal.pone.0242339)
Supplement: S1 Table — (DOCX) [file pone.0242339.s001.docx]

| **Condition** | **Value** |
| --- | --- |
| Target | 16S: V3 - V4 |
| Cycles | 29 |
| Initial | 95 *C for 7 min |
| Disassociate | 94*C for 30s |
| Anneal | 50*C for 60S |
| Extension | 72*C for 60S |
| Finish | 72*C for 7 min |
|  |  |
| Primers | 341F, 806R |
| Forward Primer (341F) | CCTAYGGGRBGCASCAG |
| Reverse Primer (806R) | GGACTACNNGGGTATCTAAT |
