# Supplementary material for: Shifts in methanogenic archaea communities and methane dynamics along a subtropical estuarine land use gradient
Source: PLoS One. 2020 Nov 24;15(11):e0242339. doi: 10.1371/journal.pone.0242339 (PMC7685437; doi:10.1371/journal.pone.0242339)
Supplement: S2 Table — (DOCX) [file pone.0242339.s002.docx]

| # of reads / ml | Site 1 | Site 2 | Site 3 | Site 4 | Site 5 | Site 6 |
| --- | --- | --- | --- | --- | --- | --- |
| Pore water | 14300 | 14743 | 8449 | 12609 | 6967 | 11692 |
| Surface water | 8137 | 6484 | 5027 | 6535 | 12054 | 12346 |
