# Supplementary figures and images for: Shifts in methanogenic archaea communities and methane dynamics along a subtropical estuarine land use gradient
Source: PLoS One. 2020 Nov 24;15(11):e0242339. doi: 10.1371/journal.pone.0242339 (PMC7685437; doi:10.1371/journal.pone.0242339)

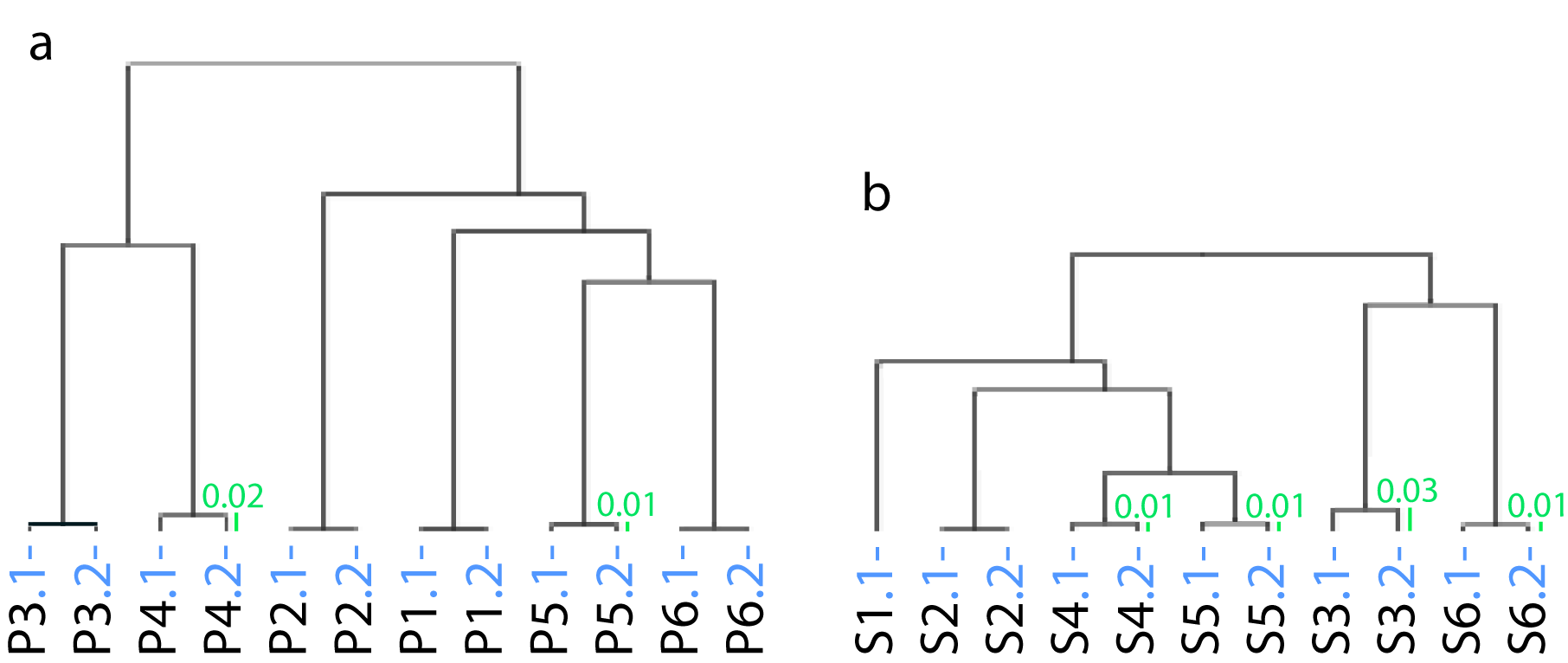

Supplement: S1 Fig — Hierarchical clustering plot using negative Pearson correlation metrics for a) pore water samples (sampling sites numbered P1 –P6) and b) surface water samples (sampling sites numbered S1 –S6); Duplicates are denoted after sample numbers in blue. Dissimilarity heights (≥ 0.01) between duplicates are shown in green. (TIF) [file pone.0242339.s003.tif]

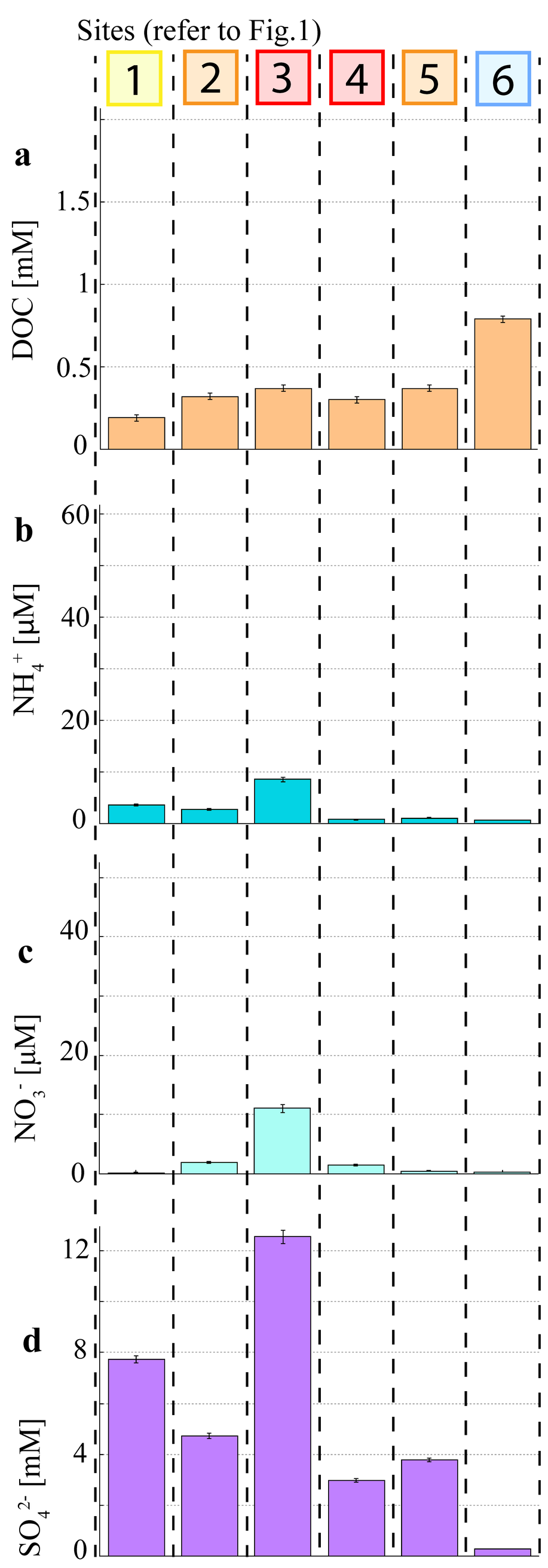

Supplement: S2 Fig — No direct links to relevant prokaryotic pore water communities. (TIF) [file pone.0242339.s004.tif]
