# Supplementary material for: Shifts in methanogenic archaea communities and methane dynamics along a subtropical estuarine land use gradient
Source: PLoS One. 2020 Nov 24;15(11):e0242339. doi: 10.1371/journal.pone.0242339 (PMC7685437; doi:10.1371/journal.pone.0242339)
Supplement: Attachment — Submitted filename: Euler_et_al_Response_to_Reviewers.docx [file pone.0242339.s008.docx]

**Manuscript: “Shifts in methanogenic archaea communities and methane dynamics along a subtropical estuarine land use gradient”**

**Response to PLOS One special issue ‘Microbial ecology of changing environments’ Reviewer’s comments:**

Note: Page/line numbers in reviewer’s comments refer to the original manuscript. Our references to page/line numbers refer to the revised manuscript.

General response: We thank the Reviewers for their constructive comments. We have made the requested amendments and addressed the comments individually as detailed below.

**Academic Editor:**

Changes of methodological description on sampling pore water, presentation of pore water data, naming of sites, improvement of the discussion as pointed out by both reviewers are required for acceptance.

Pore water sampling procedures and presentation have been clarified throughout the manuscript. Revisions have been made in accordance with Reviewer’s comments.

The collection of pore water samples has been clarified in L167-173: “At each site, discrete surface water samples (~5 cm depth) were taken from the estuary, as well as shallow groundwater / pore water samples (80 – 100 cm depth) from the immediate adjacent shoreline (within 5 meters). Shallow groundwater wells were excavated in the intertidal zone, with each well purged dry three times to ensure fresh pore water was collected. Wells were left to recharge in between sampling rounds. With this method, the signal was integrated over the whole depth range. There was little change in water table depth along the estuary and sampling sites. “

**Further amendments to pore water sampling and presentation are highlighted in the Reviewer’s comments sections.**

All site names have been adjusted to include both the site number and description: 1. estuary mouth; 2. mixed agriculture; 3. urban; 4. sewage treatment / STP; 5. bottom of the weir; 6. freshwater; with site numbers in brackets (i.e. Site 1-6) after site names, to use the same terminology rigorously throughout the manuscript.

The discussion has been improved in accordance with reviewer’s comments (**see Reviewer’s comments sections below**).

Adding qPCR data is highly recommended. Please take special care in revising the text when talking about relative abundances according to the reviewers suggestions.

The approach to relative abundances has been clarified according to reviewer’s comments. Relative abundance data was chosen to best illustrate relationships within community distributions of microbes at the different sites. We now include a table (Table S2 in the Supplementary Information) with the total number of reads per ml for each sample to give a quantitative measure for these as well. Directly relating data in the discussion via the PCA biplot has been carried out using quantitative data (absolute number of reads).

- Added in L309-313: “Notably, the total number of dsDNA detected per ml varied between samples and ranged from 5,027 at the urban site (Site 3) to 12,346 at the freshwater site (Site 6) in surface water samples and between 6,967 at the bottom of the weir (Site 5) and 14,743 at the mixed agriculture site (Site 2) in pore water samples (Table S2 in the Supplementary Information).”
- Table S2 added to the Supplementary Information (L808):

**“Table S2:** Total number of dsDNA per ml of each sample, quantified by fluorometry (see Section 2.3).

| # of reads / ml | Site 1 | Site 2 | Site 3 | Site 4 | Site 5 | Site 6 |
| --- | --- | --- | --- | --- | --- | --- |
| Pore water | 14300 | 14743 | 8449 | 12609 | 6967 | 11692 |
| Surface water | 8137 | 6484 | 5027 | 6535 | 12054 | 12346 |

“

**Further amendments are highlighted in the Reviewer’s comments sections.**

Please take special care about the description of the sampling procedure. The methods section could be improved in structure, making a distinction between sampling site, sampling procedure, environmental sample analysis, biological sample analysis and data analysis. Please add sample volumes.

The structure of the methods section has been improved and further subdivided into “2.1 Study site”, “2.2 Sample collection”, “2.3 Environmental sample analysis”, “2.4 Microbiological sample analysis” and “2.5 Data analysis”.

Environmental sample analysis has been further clarified in L198-202: “For discrete GHG samples, the gas-tight 6 L bottles were connected into a closed loop with the CRDS, with an inlet tubing bubbler used to encourage headspace equilibration. Each water sample was run for ≥ 2 h or until gas concentrations between the water sample and headspace equilibrated. GHG and nutrient concentrations were averaged for each sampling location and error propagation for measurement uncertainties applied.”

Microbiological sample analysis has been further clarified in L207-216: “Thermocycling was completed with an Applied Biosystem 384 Veriti and using Platinum SuperFi mastermix (Life Technologies, Australia) for the primary PCR. The first stage PCR was cleaned using magnetic beads, and samples were visualised on 2% Sybr Egel (Thermo-Fisher). A secondary PCR to index the amplicons was performed with TaKaRa PrimeStar Max DNA Polymerase (Clontech). The resulting amplicons were cleaned again using magnetic beads, quantified by fluorometry (Promega Quantifluor) and normalised. The equimolar pool was cleaned a final time using magnetic beads to concentrate the pool and then measured using a High-Sensitivity D1000 Tape on an Agilent 2200 TapeStation. The pool was diluted to 5nM and molarity was confirmed again using a Qubit High Sensitivity dsDNA assay (ThermoFisher). Amplicons were quantified with a dsDNA fluorometry assay (Promega Quantifluor) after two rounds of PCR.”

Sampling volumes have been added throughout the manuscript and clarified for microbiological samples in L181-184: “As water was filtered until the membrane filters’ capacity was saturated to maximize the yield of microbial tissue, total sample volumes varied from 41 ml to 129 ml for pore water samples and from 152 ml to 420 ml for surface water samples.”

**Further amendments are highlighted in the Reviewer’s comments sections.**

**Reviewer #1:**

The presented manuscript, “Shifts in methanogenic archaea communities and methane dynamics along a subtropical estuarine land-use gradient”, addresses a valuable and understudied topic. As many environmental and anthropogenic factors may influence the microbial communities, with the focus on methane related microbes, these affect the abundance of these microbes and, as a result, the methane emissions.
The authors have carried out sampling and sequencing in order to find correlations for methanogen relative abundance and the environmental parameters at different sites. It is a valuable dataset, and this manuscript has potential, although it needs some more experiments, clarity in the text, materials and methods and would certainly benefit from looking into the sequencing data in a bit more detail.

The study has one major drawback, which is the lack of qPCR data. Correlating the relative abundance of methanogens in samples with concentration measurements is not possible. When the absolute number of the total microbial community would be known, one can understand why the CH4 might be higher or lower at a site. If a percentage of the total is the only known number, one cannot compare sites in regards to abundance, whereas that would be recommendable.

Total counts per sample in the study have been evaluated but stem from fluorometry, not from qPCR as suggested by the reviewer as we were not able to conduct qPCR on the collected samples due to budget constraints. In the study, the individual amplicons were quantified using [Quantifluor](https://www.promega.com.au/products/rna-analysis/dna-and-rna-quantitation/quantifluor-dsdna-system/?catNum=E2670) fluorometry which is a dsDNA fluorometric assay that measures the concentration of each amplicon after two rounds of PCR.

We go into more detail on this, as well as the changes made to the manuscript, in the answers to the respective questions (‘Specific comments’ section).

Authors discuss methanogens and methanotrophs in terms of relative abundance, yet it remained unclear why specific parameters were present only for the surface water and not porewater, such as oxygen concentration? As the title tells, the focus is on methanogens, although the data is presented only for the surface samples and not for the porewater samples, whereas the sequencing data is available?

Pore water dissolved oxygen was not reliable due to large artefacts from sample collection in the wells. Pore water DO saturations are assumed to be close to 0 % as seen in other study with largely impermeable sediment and high amounts of organic matter.

We did measure and present sequencing data for methanogens, methanotrophs, sulfate reducers and dehalogenation bacteria (Fig. 6) as well as concentrations of DOC, NH_4_^+^, NO_3_^-^ and SO_4_^2-^ (Fig. 3) for pore water.

This is discussed in more detail, including changes made to the manuscript, in the respective parts of the ‘Specific comments’ section.

Authors have looked into the relative abundance of methanotrophs, although there was no discussion on anaerobic archaeal methanotrophs. These belong to the same order as methanogens, so the relative abundance of methanogenic orders includes microbes carrying out the reverse reaction. Suggestion is to add this information to the manuscript as then it would be complete in the methane cycle, looking at both microbial groups, methane producers, and consumers over environmental gradients as well as polluted versus more pristine sites.

We thank the reviewer for this comment. We did analyse for ANME, but found no ANME archaea in any of the samples. However, we now discuss ANME in the light of substrate and include them in the results section.

Answered in detail in the ‘Specific comments’ section (Q1 and Q6 under ‘Discussion’).

Overall comment for making it easier to read, authors use different terminology throughout the manuscript for the sites, either site 1-5, or urban and agricultural site or brackish and estuary mouth. Could the authors please choose one, as it would be easier to understand the differences between these sites?

All site names have been adjusted to include both the site number and description: 1. estuary mouth; 2. mixed agriculture; 3. urban; 4. sewage treatment / STP; 5. bottom of the weir; 6. freshwater; with site numbers in brackets (i.e. Site 1-6) after site names, to use the same terminology rigorously throughout the manuscript.

The data presentation needs polishing, as the thermal cycling of the PCR could be in the supplements and the environmental parameters for all sites shown in the manuscript itself.
Manuscript will benefit if the figures for methanogens in the porewater samples are shown the same way as for the surface water.

The table has been deleted from the main manuscript and instead moved to the supplementary material (Table S1) and referenced in the text.

Methanogen communities were present in pore water almost exclusively. The relative abundance of methanogens in surface water was <0.01%.

Clarified in more detail, as well as all changes to the manuscript, in the respective parts of the ‘Specific comments’ section.

The sequencing data should be deposited so that the analysis could be repeated on the raw data and it would be publicly available.

All raw sequencing data is now deposited in the publicly accessible database of the Knowledge Network for Biocomplexity (KNB) in the following dataset (also attached to the Supporting Information): https://knb.ecoinformatics.org/view/urn%3Auuid%3A6e145ed5-5028-4cfc-89ef-930ab2fb2bac

Comments will be addressed further in detail in the ‘Specific comments’ section below.

**#Specific comments:**

**Abstract:**

It reads as DOC concentrations were considerably higher at the freshwater site-1800uM than in the estuarine sites. Could the authors add these values?

The abstract has been changed in L35-38 to: “At the upstream freshwater site, both methane and DOC concentrations were considerably higher (2.68 μM and 1.8 mM respectively) than at the estuarine sites (0.02 to 0.66 μM and 0.39 to 1.16 mM respectively) and corresponded to the highest relative abundance of methanotrophic bacteria.“

**Materials and methods:**

Q1: Did the authors consider adding qPCR data, as the importance of relative abundance in samples depends on absolute numbers. In a sample with lower microbial abundance, the fraction assigned as methanogens does not count and remains challenging to correlate directly with the methane concentrations.

Absolute numbers are indeed very important and should have been included as a table and referenced earlier in the manuscript. They are now added as a Table with numbers of reads (normalized to reads per 1 ml) in the Supplementary Information (Table S2) and discussed more rigorously in the text.

The total counts per sample in the study do however stem from fluorometry, not from qPCR as suggested by the reviewer as we were not able to conduct qPCR on the collected samples due to budget constraints. In the study, the individual amplicons were quantified using [Quantifluor](https://www.promega.com.au/products/rna-analysis/dna-and-rna-quantitation/quantifluor-dsdna-system/?catNum=E2670) fluorometry which is a dsDNA fluorometric assay that measures the concentration of each amplicon after two rounds of PCR. Also, the PCR method & polymerases reached an excellent fidelity of x300 (measurement precision and fidelity are linked e.g. in a recent publication by Sze and Schloss [1]) and we hope that results are reflecting total abundance in the samples well.

To clarify this in the manuscript, the following changes have been made:

- Added in L214-216: “Amplicons were quantified with a dsDNA fluorometry assay (Promega Quantifluor) after two rounds of PCR.”
- In L309-313: “Notably, the total number of dsDNA detected per ml varied between samples and ranged from 5,027 at the urban site (Site 3) to 12,346 at the freshwater site (Site 6) in surface water samples and between 6,967 at the bottom of the weir (Site 5) and 14,743 at the mixed agriculture site (Site 2) in pore water samples (Table S2 in the Supplementary Information).”
- Added in L342-345: “the total number of reads from Site 6 pore water samples was 38 % higher than at the urban site (Site 3) (see Table S2 in the Supplementary Information)”
- In L484-486: “It is also noted, that total abundance measurements via PCR-based methods are generally error-prone and further research could be improved by employing additional quantification assays such as flow cytometry [1–4]”
- Table S2 added to the Supplementary Information (L808):

**“Table S2:** Total number of dsDNA per ml of each sample, quantified by fluorometry (see Section 2.3).

| # of reads / ml | Site 1 | Site 2 | Site 3 | Site 4 | Site 5 | Site 6 |
| --- | --- | --- | --- | --- | --- | --- |
| Pore water | 14300 | 14743 | 8449 | 12609 | 6967 | 11692 |
| Surface water | 8137 | 6484 | 5027 | 6535 | 12054 | 12346 |

“

Q2: 170-172: could the authors clarify how the porewater samples were taken? Was the surface water taken from the top~5cm, and immediately porewater at the same location from the bottom? Or sampled at a different location, closer to the shore?
How much did the water column hight vary along the estuary and the sampling sites?

Surface water samples were sampled from river water adjacent to the shore (within 5 meters; at ~5 cm depth); pore water was sampled from shallow wells at 80 – 100 cm depth at the shore. Wells were left to recharge in between sampling rounds. This way, the signal was integrated over the whole depth range. Depths did not vary much at the stated proximity to the shore.

This was clarified in the manuscript in L167-173: “At each site, discrete surface water samples (~5 cm depth) were taken from the estuary, as well as shallow groundwater / pore water samples (80 – 100 cm depth) from the immediate adjacent shoreline (within 5 meters). Shallow groundwater wells were excavated in the intertidal zone, with each well purged dry three times to ensure fresh pore water was collected. Wells were left to recharge in between sampling rounds. With this method, the signal was integrated over the whole depth range. There was little change in water table depth along the estuary and sampling sites.”

Q3: L181: Were the tubes pre-filled with DNAgard?

Yes, tubes were pre-filled under sterile conditions prior to departure.

Clarified in the manuscript in L179-181: “Microbial samples were filtered with 0.22 μm membrane filters (Isopore) and stored in sterile 1.5 ml polycarbonate vials (Eppendorf) pre-filled with DNAgard (Sigma) under the flow hood maintaining sterile conditions.”

Q4: L182: What is the relevance of the 2 min interval in sampling? For replication, to ensure the sampling of the community and water samples at the exact time point?

Yes this is correct, the 2 min interval refers to duplicate sampling to ensure temporal congruency. To clarify this in the manuscript, we added in L184-185: “In order to gather duplicates, sampling took place within 2 minutes of each other at each site”

Q5: L200: Could the authors clarify these steps or refer to a protocol supplied by the sequencing facility.

A variation of the Illumina 16S metagenomics sequencing protocol has been used by the sequencing facility. For the protocol we were referred to Illumina Technical Support [5].

To clarify this in the manuscript, we added in L218-219: “A variation of the Illumina 16S metagenomics sequencing protocol was utilized for this purpose.”

L214: The sentence does not read well, replace clustered followed with clustered before/prior chimera filtering.

The sentence was changed to improve readability in L226: “Sequences were clustered before chimera filtering”

L215: Check for double spacing.

Double spacing removed.

Q6: L219-220: What do authors mean by sampling volume?

As microorganisms were collected on 0.22 um filters, the total number of cells correlated directly to the amount of water filtered through the filters. To maximize the yield, water was filtered until the filter papers clogged. This resulted in varying amounts of sampling water volume and the number of reads were thus subsequently normalized (to 1 ml).

To clarify this in the manuscript, we added in L181-184: “As water was filtered until the membrane filters’ capacity was saturated to maximize the yield of microbial tissue, total sample volumes varied from 41 ml to 129 ml for pore water samples and from 152 ml to 420 ml for surface water samples.“ and in L231-232: “As sample volumes varied, the number of reads were normalized per 1 ml prior to analysis.”

**Results:**

Q1: The presented data for oxygen, methane etc., is presented for surface water. Is there the same data available for porewater samples? It would be easier for the reader if both datasets are presented within the manuscript and not as a figure in the manuscript and a table with additions in the supplementary table.

A) Pore water dissolved oxygen was not reliable due to large artefacts from sample collection in the wells. However, we can assume that DO saturation would be close to 0%.

To clarify this in the manuscript, we added in L259-261: “Pore water DO saturations are assumed to be close to 0% as seen in other study with largely impermeable sediment and high amounts of organic matter [6–8]”

B) Gas measurements were only carried out at a constant depth below the surface as only one cavity ring down spectrometer was present and the whole boat side construction we devised did not measure higher/variable depths and/or pore water.

Added in L288-290: “Both CH_4_ and CO_2_ concentrations have only been measured in the water column at a fixed depth (0.5 – 0.7 m) due to limitations in the sampling apparatus. Additional oxidation of gas fluxes prior to reaching the water column sampling location thus need to be considered for pore water processes.”

C) We did measure and present sequencing data for methanogens, methanotrophs, sulfate reducers and dehalogenation bacteria (Fig. 6) as well as concentrations of DOC, NH_4_^+^, NO_3_^-^ and SO_4_^2-^ (Fig. 3) for pore water.

Q2: The presented prokaryotic community data is discussed in section 3.3. Is that for porewater or surface water?

This was not made very clear in the text. Communities were isolated from the data set for either pore or surface water, depending on their function and relative abundance. Correspondingly, the relative abundance of methanogens in surface water was <0.01%. Sampling locations are depicted in Figure 6.

Clarified in L344-345: “Methanogenic archaea detected in surface water were below 0.01 % (or 25 reads) and thus not further considered.”

Q3: On figures is presented the methanogen relative abundance in the porewater, could the authors add the same figure/data for surface water?

The relative abundance of methanogens in surface water was <0.01% (or <25 reads), see Q2.

Clarified in L344-345: “Methanogenic archaea detected in surface water were below 0.01 % (or 25 reads) and thus not further considered.”

Q4: Methanogen relative abundance is high at the sites with higher methane concentrations as well as higher DOC concentration. However, methanogens are considered mostly as anaerobic microorganisms. Could the authors comment on these correlations, especially with oxygen?

As methanogens appear exclusively in pore water, where assumedly DO concentrations are low due to high organic carbon concentrations, anaerobic conditions are interpreted as a precursor for the correlations between methanogens, CH4 and DOC. I.e. when electron acceptors with high redox potentials are depleted (especially oxygen) and enough carbon sources (DOC) are present, methanogens proliferate and CH4 is produced. These conditions occur in the urban environment.

Pore water DO concentrations were not measured, but assumed to be close to zero, as clarified in Q1.

Clarified anoxic pore water in L336-339: “Methanogenic archaea in anoxic pore water samples showed a higher relative abundance at the urban site (Site 3) and sewage treatment plant (Site 4) as well as the freshwater site (Site 6). Relative abundances of pore water methanogenic archaea ranged from 0.2 % at the mixed agriculture site (Site 2) to 7.8 % near the sewage treatment plant (Site 4).”

L239-240: It is subjective. For some microbes, a 2C difference might not be small.

Good point. We have now adjusted the sentence in L252-253 to: “Water temperature showed a maximum of 29.4 °C at the estuary mouth (Site 1) and a minimum of 27.4 °C at the mixed agriculture site (Site 2). “

L242: Remove minor.

Minor removed.

L251: In the abstract, the values are presented on the micromolar scale. For consistency throughout the manuscript, please use the same units. It makes it easier for the reader.

Thanks for pointing that out. GHG concentration units are now presented in units of micromolar throughout the manuscript.

Q5: L321-323: Could the authors add the same data for methanogens?

The relative abundance of methanogens was <0.01% (or <25 reads), see Q2 & Q3. All sequencing data is now also attached to the publication as raw data and a table included (Table S2 in the Supplementary Information) with total number of reads per ml for each sample.

Added in L344-345: “Methanogenic archaea detected in surface water were below 0.01 % (or 25 reads) and thus not further considered.”

**Discussion:**

Q1: In case there was a high relative abundance of methanogens at sites 3 and 4, as well as more DOC, what feeds this reaction? The discussion could go more in depth in this topic, as humic substances are part of DOC, and have shown to suppress/enhance methanogenesis, whereas simultaneously serve as the electron acceptor for anaerobic methanotrophs (ANME archaea).

We would assume that the reaction is realized by coexisting specialized microorganisms with varying metabolic pathways, e.g. in this case decomposers would feed on and transform DOC molecules into CO_2_, which is the most common electron acceptor used by methanogens. We did not, however, differentiate between different DOC molecules as this was beyond the scope of our study. That is valuable point though - differences in the communities may occur due to different types of DOC molecules. Humic substances are very well suited to demonstrate this. A low concentration of the latter could be inferred from our results because large fractions of methanogens and sulfate reducers were present in the samples (both inhibited by humic substances). Conversely no ANME were detected.

To clarify this in the manuscript, we added in L440-443: “These results also point to a low concentration of humic substances in the DOC, which have been shown to inhibit both methanogenesis and sulfate reduction as well as serve as an electron acceptor for ANME (absent in our samples; see section 3.3) [9–11].”

Q2: As there is a difference between sites 3 and 4 in the oxygen content, although not much lower methane concentrations. One could think that anaerobic/less oxic conditions would make methanogens thrive. How can this be explained or how less oxic conditions affect the distribution of the overall community? Would more taxonomic groups of fermenting microbes increase?

We agree the microbial community distributions are closely linked with oxygen concentrations as oxygen is the final electron acceptor in oxidative phosphorylation with the highest redox potential. As such, the interplay between nutrient and/or electron acceptor availability and microbial communities is likely the foundation of observed phenomena. Additionally, in the end it also depends on competition between individual taxa, whether we see communities that utilize other electron acceptors like SO_4_^2-^, NO_3_^-^, CO_2_, etc, or fermenters that use organic molecules. Fermenters would also produce hydrogen, which methanogens can use as electron donors.

To clarify the limitation of methanogens to anoxic pore water in the discussion we added in L392-394: “Conversely, aerobic conditions in the surface water (DO concentrations of 26.6% to 106.4%; Fig. 2) limit methanogen communities to the anoxic pore water.”

We also added in L419-422: “As there is almost no sulfate (Fig. 3) and a low relative abundance of sulfate reducers (Fig. 6) present at the freshwater site (Site 6), a lack of competition for hydrogen produced by fermenters in the anoxic pore water could explain higher methane production rates of methanogens which commonly use H_2_ as an electron donor [12,13]”

Q3: Lines 375-377, it is not possible to relate a percentage of sequencing data with the methane concentration. One would need to know the absolute number or retrieve that from the absolute copy numbers quantified with the same primers with what the sequencing was carried out. Could the authors explain how concentration can be correlated with a percentage of microbes in this dataset?

Thank you for pointing out the apparent relation of quantitative (GHG concentrations) and semi-quantitative (relative abundances) data in the text. Relative abundance data was chosen to best illustrate relationships within community distributions of microbes at the different sites. We now include a table (Table S2 in the Supplementary Information) with the total number of reads per ml for each sample to give a quantitative measure for these as well. Directly relating data in the discussion via the PCA biplot has been carried out using quantitative data (absolute number of reads).

Q4: The authors found Methylacidiphilales order, yet these microbes are mainly found in very acidic sites with pH down to 2. In extreme conditions with high temperatures, what could be the role of these in an estuary system?

Thanks for the interesting input. We can only assume that micro-environments are present that allow for acidophilic bacteria to proliferate when the thermodynamic energy landscape is right (e.g. enough CH4 to oxidize). As to why they are only appearing in the freshwater site (Site 6) is indeed very curious and we can’t explain this phenomenon. A potential lack of minerals acting as a pH buffer in the water may contribute by facilitating larger variations in pH between micro-environments.

Added in L476-477: “The potential existence of distinct microenvironments entailing different pH could explain the prevalence of acidophilic *Methylacidiphilales* at the freshwater site [14,15].”

Q5: Has this order of Methylacidiphilales been found in previous studies in estuaries? The found sequences in this order clustered all together or showed variations at lower levels? Could the authors go down to the genus level?

Only the order of *Methylacidiphilales* and respective family of *Methylacidiphilaceae* were found. We did not detect further variation in the sequences at lower levels. We are not aware of any studies discovering *Methylacidiphilales* in estuaries specifically.

Q6: Could the authors include a discussion on anaerobic methanotrophs in case these were present in the data? It would be beneficial to find the literature on ANME archaea, look into salt and freshwater differences, and their contribution.

ANME were targeted within the 16s sequencing pipeline, but no ANME archaea were found in any of the samples.

The lack of discovered ANME archaea is now stated in the Results in L351-352: “All detected methanotrophic prokaryotes belonged to the bacteria kingdom with no ANME (anaerobic methanotrophic archaea) found in any of the samples. “

**Reviewer #2:**

The manuscript by Euler et al entitled “Shifts in methanogenic archaea communities and methane dynamics along a subtropical estuarine land use gradient” describes the effect of changes in physiochemical, nutrient, and greenhouse gas distributions on the microbial communities in an estuary. Specifically, anthropogenic altered environments were compared to more pristine sites within the estuary. DOC, methane, O2, SO4, etc. were measured in surface in pore water samples and correlated with microbial identification and abundances using 16s rRNA. Overall, the manuscript is clear and concise. The conclusions are largely supported by the data. There are no apparent technical issues. However, the results are largely confirmatory of previous studies and expected; environments with higher DOC and scarce thermodynamically favorable electron acceptors have increased methanogenesis (more methanogens), which in turn can support methanotrophs. Urban and sewage treatment plant sites would be expected to result in increased DOC etc. This limits the significance of the study.

We thank the reviewer for their constructive comments. We believe the study makes a significant and novel contribution to the literature on how microbial communities drive methane dynamics along an estuarine continuum. This was the first study to combine the evaluation of methanogen communities in altered estuarine environments together with high resolution GHG data, and although some results may be expected intuitively, empirically producing evidence of the nature of these interconnections is an important part of the scientific method. Furthermore, sequencing data does not solely verify fundamental thermodynamic models produced in the lab, but also elucidates these models at environmental scales, adding to the growing body of data about what specific microbial players communities are made up of. For example, prokaryotes build an intricate web of individual microorganisms, including various methane cyclers, which are all present at different magnitudes and constituted by different taxa. All of which are significant data points to ultimately understand the microbial side of important downstream effects caused by anthropogenic impact on these environments as well as resulting greenhouse gas emission dynamics.

All other comments will be addressed in the ‘Specific comments’ section below.

**###Specific comments:**

**1. Abstract lines 37-39:** This sentence is confusing as written. It implies a comparison SRBs to electron acceptors. Possible changing “whilst” to “consistent”

The sentence has been changed in L38-41 to: “The proportion of sulfate reducing bacteria in the prokaryotic community was elevated within the urban and STP sites (relative abundances of 8.0% – 10.5%), consistent with electron acceptors with higher redox potentials (e.g. O_2_, NO_3_^-^) being scarce.”

**2. Lines 98-105 (and other sections):** As written, this paragraph implies methane consumption is only performed by bacteria, which of course is not true. Anaerobic methanotrophic archaea (e.g. ANME) contribute to methane consumption. Were ANME targeted or detected in this environment? or considered to play a role? It would be good to mention their existence at the very least.

Yes, thanks for pointing this out. ANME were targeted within the 16s sequencing pipeline, however none were detected.

This has been clarified in L351-352: “All detected methanotrophic prokaryotes belonged to the bacteria kingdom with no ANME (anaerobic methanotrophic archaea found in any of the samples.”

**3. Table 1:** I do not believe this information is necessary in a table format. It should simply be listed in the methods.

Consistent with Reviewer#1’s suggestions the table has been deleted from the main manuscript and instead moved to the supplementary material and referenced in the text.

We have added in L206-207: “PCR amplification and sequencing were performed at the Australian Genome Research Facility using the primers and conditions outlined in Table S1 in Supplementary Information.”

**4. Lines 286-289:** It is stated that 6 of 7 methanogen orders were detected, it seems figure 6 should be referenced here, or this sentence moved to the next section.

‘Fig. 6’ is now referred to in the text.

**5. Results title 3.3 “Methanogenic archaea and environmental OTUs”:** This section discusses more than methanogenic archaea, so it seems odd that methanogenic archaea are specifically stated.

This is indeed a little confusing. The section title has now been changed to “3.3 Environmental OTUs”.

**6. Line 324-325 and Fig. 6:** Is site 6 really dominated by Methylacidiphilales? Based on the bar graph in Fig. 6 it looks like Methylococcales are a higher proportion. Also, due to the very low abundance of methyltrophs in the other sites, Figure 6 does not support the conclusion that type I and type II methylotrophs dominate the other sites, since the bars are too small.

We agree, this is unclear in the manuscript. Since methanotrophs were present in both surface and pore water samples, we included bar plots for both. Methylacidiphilales dominated only the site 6 surface water samples, while pore water samples were indeed dominated by Methylococcales & Methylocystaceae. To reflect that Methylacidiphilales dominated specifically only one surface water site (Site 6) and type I & II methanotrophs did not dominate the other sites, the sentence was rewritten in L348-351: “Site 6 surface water samples were the only ones dominated by the thermoacidophilic methanotroph order *Methylacidiphilales*, with only a low fraction of type I or type II methanotrophs. The relative abundance of methanotrophs was below 0.5 % at all other sites.”

**7. Lines 387-390:** It seems unlikely that differences in methane production rates between orders accounts for the differences in observed CH4 concentrations, and why high in site 6. I do not believe there is evidence for large differences in methane production under similar conditions. Substrate concentration and other environmental factors are largely responsible for methane production rates, not differences between individual species. It seems more likely that CH4 is highest in site 6, compared to the others, simply because there is far less sulfate. Methanogens at this site can outcompete SRBs for hydrogen produced by fermenting bacteria, where at all other sites sulfate is prevalent.

We agree that differences in the substrate, i.e. more available hydrogen due to a lack of competition in the case of freshwater site 6, can play a large role in observed differences in methane production. However, we don’t know how likely or unlikely it is that different taxa could also contribute to differences in CH_4_ concentrations as well. There was higher methane concentrations in the fresh water sites which is not reflected in the overall abundance of methanogen communities.

In accordance with Reviewer#2’s suggestion, we included competition for hydrogen as an explanation for observed methane production differences before variations in methanogen communities; in L419-422: “As there is almost no sulfate (Fig. 3) and a low relative abundance of sulfate reducers (Fig. 6) present at the freshwater site (Site 6), a lack of competition for hydrogen produced by fermenters in the anoxic pore water could explain higher methane production rates of methanogens which commonly use H_2_ as an electron donor [12,13].”

Also, many Methanobacteriales and Methanomicrobiales, which are the most abundant in this study, have been cultured.

Yes, small fractions can be cultured but don’t represent diverse natural communities.

Sentence changed in L425-427: “The quantification of methane production rates of different methanogenesis taxa is still lacking in the literature owing to difficulties in culturing most of these microorganisms”

Finally, what about Methanosarcinales? They were highly abundant in the sites with the most CH4 (4 and 6). Methanosarcinales are the most metabolically diverse methanogens and thrive in both freshwater and saltwater environments. In fact, in site 6 it appears Methanosarcinales were far more prevalent than Methanobacteriales. It is surprising that this was not addressed, but it should be.

The fraction of *Methanobacteriales* within methanogen communities increased much more drastically than other taxa in Site 6 (from ~3-8% to ~20%). That was the observation we were highlighting here. *Methanosarcinales* should indeed also be discussed though, thank you for pointing this out.

Added in L405-407: “The metabolically diverse *Methanosarcinales* showed a high relative abundance in pore water samples at all sites where methane concentrations were high (between 41% and 68% of total methanogen communities at Sites 3, 4 and 6).”

**8. Line 437-438:** This is not a valid statement in the opinion of this reviewer. Aerobic methanotrophic bacteria are well characterized. The genes, enzymes, pathways, regulation etc. have all been studied. Also, it seems unlikely again that rates of methane oxidation (lines 435-437) is a valid argument for the observed differences.

We agree, aerobic methanotrophs have been well studied microbiologically. However, data sets of methane cycler community compositions in situ and in the light of different environmental parameters are still lacking and there are few studies for estuaries. We suggest that the latter is an important step in the scientific method and to gauge downstream effects of anthropogenic impact specifically.

We also agree that that different rates of methane oxidation between taxa cannot be concluded from our data. However, if more methane is oxidized per taxon it would certainly contribute to differences in methane concentrations, which is ultimately all we can hypothesize based on our findings.

In order to clarify this in the manuscript, we made the following changes in L465-467: “Varying rates of CH_4_ oxidation in different taxa could be hypothesized to contribute to observed differences in CH_4_ concentrations.”

**References:**

1.	Sze MA, Schloss PD. The Impact of DNA Polymerase and Number of Rounds of Amplification in PCR on 16S rRNA Gene Sequence Data. mSphere. 2019;4. doi:10.1128/mSphere.00163-19

2.	Smith CJ, Osborn AM. Advantages and limitations of quantitative PCR (Q-PCR)-based approaches in microbial ecology. FEMS Microbiol Ecol. 2009;67: 6–20.

3.	Props R, Kerckhof F-M, Rubbens P, De Vrieze J, Hernandez Sanabria E, Waegeman W, et al. Absolute quantification of microbial taxon abundances. ISME J. 2017;11: 584–587.

4.	Özel Duygan BD, Hadadi N, Babu AF, Seyfried M, van der Meer JR. Rapid detection of microbiota cell type diversity using machine-learned classification of flow cytometry data. Commun Biol. 2020;3: 379.

5.	Illumina. Technical Support: 16s metagenomic sequencing library preparation guide. 2013. Available: https://support.illumina.com/downloads/16s_metagenomic_sequencing_library_preparation.html

6.	Glud RN. Oxygen dynamics of marine sediments. Mar Biol Res. 2008;4: 243–289.

7.	Probandt D, Knittel K, Tegetmeyer HE, Ahmerkamp S, Holtappels M, Amann R. Permeability shapes bacterial communities in sublittoral surface sediments. Environ Microbiol. 2017;19: 1584–1599.

8.	Higashino M. Quantifying a significance of sediment particle size to hyporheic sedimentary oxygen demand with a permeable stream bed. Environ Fluid Mech. 2013;13: 227–241.

9.	Bai Y-N, Wang X-N, Wu J, Lu Y-Z, Fu L, Zhang F, et al. Humic substances as electron acceptors for anaerobic oxidation of methane driven by ANME-2d. Water Res. 2019;164: 114935.

10.	Vigneron A, Cruaud P, Pignet P, Caprais J-C, Cambon-Bonavita M-A, Godfroy A, et al. Archaeal and anaerobic methane oxidizer communities in the Sonora Margin cold seeps, Guaymas Basin (Gulf of California). ISME J. 2013;7: 1595–1608.

11.	Wang F-P, Zhang Y, Chen Y, He Y, Qi J, Hinrichs K-U, et al. Methanotrophic archaea possessing diverging methane-oxidizing and electron-transporting pathways. ISME J. 2014;8: 1069–1078.

12.	Sela-Adler M, Ronen Z, Herut B, Antler G, Vigderovich H, Eckert W, et al. Co-existence of Methanogenesis and Sulfate Reduction with Common Substrates in Sulfate-Rich Estuarine Sediments. Front Microbiol. 2017;8: 766.

13.	Egger M, Lenstra W, Jong D, Meysman FJR, Sapart CJ, van der Veen C, et al. Rapid Sediment Accumulation Results in High Methane Effluxes from Coastal Sediments. PLoS One. 2016;11: e0161609.

14.	Trampe ECL, Larsen JEN, Glaring MA, Stougaard P, Kühl M. In situ Dynamics of O2, pH, Light, and Photosynthesis in Ikaite Tufa Columns (Ikka Fjord, Greenland)-A Unique Microbial Habitat. Front Microbiol. 2016;7: 722.

15.	Gieseke A, Tarre S, Green M, de Beer D. Nitrification in a biofilm at low pH values: role of in situ microenvironments and acid tolerance. Appl Environ Microbiol. 2006;72: 4283–4292.
